# Equivalent Circuit Model of Low-Frequency Magnetoelectric Effect in Disk-Type Terfenol-D/PZT Laminate Composites Considering a New Interface Coupling Factor

**DOI:** 10.3390/s17061399

**Published:** 2017-06-15

**Authors:** Guofeng Lou, Xinjie Yu, Shihua Lu

**Affiliations:** State Key Lab of Power System, Department of Electrical Engineering, Tsinghua University, Beijing 100084, China; lgf13@mails.tsinghua.edu.cn (G.L.); sh-lu14@mails.tsinghua.edu.cn (S.L.)

**Keywords:** magnetoelectric effect, magnetoelectric laminate composite, equivalent circuit model, interface coupling factor, magnetoelectric laminate based sensor

## Abstract

This paper describes the modeling of magnetoelectric (ME) effects for disk-type Terfenol-D (Tb_0.3_Dy_0.7_Fe_1.92_)/PZT (Pb(Zr,Ti)O_3_) laminate composite at low frequency by combining the advantages of the static elastic model and the equivalent circuit model, aiming at providing a guidance for the design and fabrication of the sensors based on magnetoelectric laminate composite. Considering that the strains of the magnetostrictive and piezoelectric layers are not equal in actual operating due to the epoxy resin adhesive bonding condition, the magnetostrictive and piezoelectric layers were first modeled through the equation of motion separately, and then coupled together with a new interface coupling factor *k*_c_, which physically reflects the strain transfer between the phases. Furthermore, a theoretical expression containing *k*_c_ for the transverse ME voltage coefficient *α*_v_ and the optimum thickness ratio *n*_optim_ to which the maximum ME voltage coefficient corresponds were derived from the modified equivalent circuit of ME laminate, where the interface coupling factor acted as an ideal transformer. To explore the influence of mechanical load on the interface coupling factor *k*_c_, two sets of weights, i.e., 100 g and 500 g, were placed on the top of the ME laminates with the same thickness ratio *n* in the sample fabrication. A total of 22 T-T mode disk-type ME laminate samples with different configurations were fabricated. The interface coupling factors determined from the measured *α*_v_ and the DC bias magnetic field *H*_bias_ were 0.11 for 500 g pre-mechanical load and 0.08 for 100 g pre-mechanical load. Furthermore, the measured optimum thickness ratios were 0.61 for *k*_c_ = 0.11 and 0.56 for *k*_c_ = 0.08. Both the theoretical ME voltage coefficient *α*_v_ and optimum thickness ratio *n*_optim_ containing *k*_c_ agreed well with the measured data, verifying the reasonability and correctness for the introduction of *k*_c_ in the modified equivalent circuit model.

## 1. Introduction

The magnetoelectric (ME) effect is defined as a dielectric polarization of a material when a magnetic field is applied, or, conversely, a magnetization of a material when an electric field is applied [1,2,3]. The intrinsic ME effect was first observed in single-phase material by Curie in 1894 [4]. Due to its high requirement for the temperature and low inherent magnetoelectric coupling, little research has been done on single phase ME material. Since van Suchtelen proposed the concept of a product property in the composite combining magnetostrictive and piezoelectric phases in 1972 [5,6,7], the large extrinsic ME effects in bulk composites have drawn great attention. The development of bulk ME composites reached a milestone in 2001, when the giant ME effect was reported in the Terfenol-D/PZT laminate composite by Ryu et al. [8,9,10].

In the past two decades, researchers have focused on developing analytical models to explain and predict the ME effect in ME laminate composites. The first theoretical analysis was performed by Harshe et al. in 1991 [11,12,13]. They proposed a simple static elastic model, in which the magnetic and piezoelectric layers were assumed to be perfectly bonded and the strains in the both layers along the transverse directions were equal. Compared to this simple model, Nan developed a rigorous Green’s function technique to solve the constitutive equations [14,15], which is universal to the ME composites with various connectivity, not only 2-2 type laminate composite. By applying this method, all effective composite properties could be derived, but it could hardly provide a simple expression for predicting the ME coefficient.

The models proposed by Harshe and Nan both supposed the interface between the phases to be ideally coupled, which does not exist in the actual condition. On this basis, Bichurin et al. proposed another generalized static elastic model [16,17] to calculate the effective ME coefficient at low frequency by introducing an interface coupling *k*, which represents the actual bonding condition between the magnetic and piezoelectric layers. In their modeling, the laminate composite was considered as a homogeneous bilayer whose effective material parameters in the constitutive equations were estimated by an averaging method, which is similar to the Green’s function. However, the calculation and expression for the effective ME coefficient was still complicated, although much simpler than Green’s function technique. Filippov agreed the coupling between the layers was not ideal, but suggested that the laminate composite could not be regarded as a homogeneous medium [18]. The definition of coupling coefficient between the phases proposed by Bichurin was also applied in his theory of ME effect for heterogeneous composites. Martins et al. defined a similar interface coupling factor for the polymer based composites and further proposed a ME coupling model for the particulate composites [19,20,21].

However, the above modeling methods are all applicable for static or quasi-static magnetic field, while the energy transduction of ME laminate composite commonly undergoes dynamic drive. Dong et al. proposed an equivalent circuit method account for ME effect under dynamic magnetic field drive (quasi-static condition as well), in which the magnetostrictive and piezoelectric layers were coupled though an equation of motion [22,23]. Although the theory provides simpler calculation and ME coefficient expression than the static elastic methods, it considered the laminate composite as a homogeneous medium and assumed that the magnetostrictive and piezoelectric layers have the same displacement and strain, i.e., the interface between layers were perfect. Consequently, the ME coefficient derived from the equivalent circuit could not reflect the actual interface coupling condition. 

In brief, Bichurin’s generalized elastic model considered the inherent imperfect coupling interface but got a complicated expression for ME coefficient for static or quasi-static magnetic field, while Dong’s equivalent circuit model provided a simple and straightforward ME prediction for dynamic situations but ignored the actual interface coupling condition.

To overcome their drawbacks and combine their advantages, a modified equivalent circuit model considering the newly proposed interface coupling factor, under quasi-static and dynamic magnetic field drive, is proposed to predict the ME coefficient of 2-2 type laminate composite in this paper. Furthermore, the previous studies on the ME laminate composite are all based on long-type configuration. It is necessary to take the other configurations, such as disk-type, into consideration. This paper focuses on the modeling of disk-type ME laminate composite and aims to provide guidance for the design, fabrication and application of the ME laminate based devices, such as current sensor, magnetic sensor, energy harvester and wireless energy transfer system.

## 2. Theoretical Analysis 

**Figure 1:**
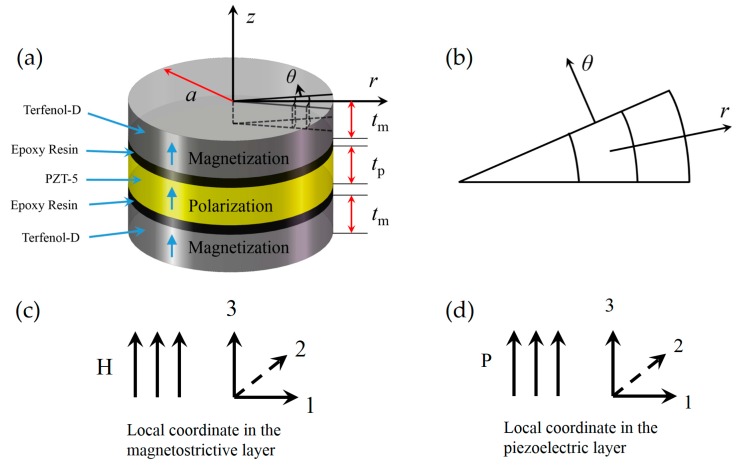
(**a**) The schematic of the T-T mode disk-type ME laminate composite in the cylindrical coordinate, which consists of a piezoelectric PZT-5 layer poled in its transverse direction sandwiched between two magnetostrictive Terfenol-D layers magnetized in their transverse direction. The thicknesses of the magnetostrictive and piezoelectric layer are *t*_m_ and *t*_p_, respectively. Their radii are both *a*. The dashed volume represents a micro sector ring shaped mass unit. (**b**) The schematic of a micro sector ring shaped mass unit. (**c**) The local coordinate in the magnetostrictive layer, in which 3 denotes the magnetization direction. (**d**) The local coordinate in the piezoelectric layer, in which 3 denotes the polarization direction.

Figure 1a shows the schematic of T-T mode disk-type laminate composite, which consists of a piezoelectric PZT-5 layer poled in its thickness (or transverse) direction sandwiched between two magnetostrictive Terfenol-D layers magnetized in their thickness (or transverse) direction. The thicknesses of the magnetostrictive and piezoelectric layer are *t*_m_ and *t*_p_, respectively. Their radii are both *a*. The dashed volume represents a micro sector ring shaped mass unit. For the convenience of modeling, the diameter of each layer is assumed much larger than its thickness and the thickness of the epoxy resin layers could be ignored, i.e., the laminate could be regarded as an in-plane, two-dimensional disk-type laminate. 

It also should be noticed that the cylindrical coordinate system is applied in the theoretical analysis due to the disk-type configuration. For a micro sector ring shaped mass unit in the disk shown in Figure 1a,b, *r* axis is along the radial direction, *θ* axis is vertical to the edge, and *z* axis is along the axial direction.

For a given geometrical dimension, the main magnetostrictive deformation is along the maximum dimension direction of the sample. When an AC magnetic field *H*_ac_ is applied along the thickness direction (denoted 3 in the local coordinate of magnetostrictive layer in Figure 1c), the magnetostrictive layers stretch and shrink along the main strain (or vibration) direction, i.e., the radial direction. Through the bonding of epoxy resin, the piezoelectric layer is forced into oscillation along the same direction, which excites a radial symmetric vibration mode, generating a voltage across the thickness direction (denoted 3 in the local coordinate of piezoelectric layer in Figure 1d) due to the transverse polarization of the piezoelectric layer. 

Therefore, the working mechanism of the T-T mode ME laminate could be physically divided into three steps: the radial vibration of the magnetostrictive layer, the transfer of the strain, and the radial vibration of the piezoelectric layer. Except Bichurin’s group, previous researchers have paid more attention to the magnetostrictive and piezoelectric constitutive equations, rather than the strain transfer between them, which is mostly dependent on the interface coupling condition in strain-mediated bulk ME laminate composite. Dong et al. ignored the losses in the strain transfer and assumed the strains of the magnetostrictive and piezoelectric layers to be equal [22], resulting in the discrepancy between the theoretical and experimental data. Although a rectifying factor was empirically introduced to adjust the discrepancy [24], it lacked a realistic physical meaning. Essentially, the strains of both layers are not equal in actual working condition. In our analysis, the vibration and strains of the magnetostrictive and piezoelectric layers are first modeled through the equation of motion separately [25,26,27,28], and then coupled together with a new interface coupling factor that physically reflects the strain transfer between the phases, which is different from the definition by Bichurin et al. [17].

### 2.1. Equivalent Circuit of Magnetostrictive Layer

Terfenol-D is a typical ferromagnet with the cubic symmetry. When *H*_ac_ is applied along the z-axis, a radial symmetric vibration is excited in the plane of Terfenol-D layer. The corresponding constitutive equations consist of nonzero terms for the cubic symmetry are

(1a) $$S_{rm}=s_{11}^{H}T_{rm}+s_{12}^{H}T_{\theta m}+d_{31,m}H_{z},$$

(1b) $$S_{\theta m}=s_{12}^{H}T_{rm}+s_{11}^{H}T_{\theta m}+d_{31,m}H_{z},$$

(1c) $$B_{z}=d_{31,m}T_{rm}+d_{31,m}T_{\theta m}+\mu_{33}^{T}H_{z}.$$

where the subscript m denotes the magnetostrictive layer. $S_{rm}$ and $S_{\theta m}$ are the strain along the radial and azimuth direction, respectively. $T_{rm}$ and $T_{\theta m}$ are the stress along the radial and azimuth direction, respectively. $H_{z}$ and $B_{z}$ are the magnetic field and magnetic flux density along the thickness direction. $s_{11}^{H}$ and $s_{12}^{H}$ are the compliance coefficients at constant *H*. $d_{31,m}$ is the transverse piezomagnetic coefficient, and $\mu_{33}^{T}$ is the permeability at constant stress along the axial direction. The constitutive equations can be rewritten in terms of the stress and magnetic flux density as

(2a) $$T_{rm}=\frac{1}{s_{11}^{H}(1-\sigma_{m}^{2})}(S_{rm}+\sigma_{m}S_{\theta m})-\frac{d_{31,m}}{s_{11}^{H}(1-\sigma_{m})}H_{z},$$

(2b) $$T_{\theta m}=\frac{1}{s_{11}^{H}(1-\sigma_{m}^{2})}(S_{\theta m}+\sigma_{m}S_{rm})-\frac{d_{31,m}}{s_{11}^{H}(1-\sigma_{m})}H_{z},$$

(2c) $$B_{z}=d_{31,m}(T_{rm}+T_{\theta m})+\mu_{33}^{T}H_{z}.$$

where $\sigma_{m}=-\frac{s_{12}^{H}}{s_{11}^{H}}$ is the Possion ratio of Terfenol-D. According to the Newton’s second law, the radial equation of motion for the Terfenol-D layer is

(3) $$\frac{\partial T_{rm}}{\partial r}+\frac{T_{rm}-T_{\theta m}}{r}=\rho_{m}\frac{\partial^{2}u_{rm}}{\partial t^{2}},$$

where $\rho_{m}$ and $u_{rm}$ are the density and displacement of Terfenol-D layer, respectively. By substituting Equations (2a) and (2b) into Equation (3), and assuming that the magnetic field is evenly distributed along the radial direction, i.e., $\partial H_{z}/\partial r=0$, and using the definition of strain

(4) $$S_{rm}=\frac{\partial u_{rm}}{\partial r},S_{\theta m}=\frac{u_{rm}}{r},$$

the radial equation of motion can be rewritten as ($u_{rm}$ is simplified as $u_{m}$ for convenience)

(5) $$\;\frac{\partial^{2}u_{m}}{\partial r^{2}}+\frac{1}{r}\frac{\partial u_{m}}{\partial r}-\frac{u_{m}}{r^{2}}=\frac{1}{\upsilon_{m}^{2}}\frac{\partial^{2}u_{m}}{\partial t^{2}},$$

where $\upsilon_{m}=\sqrt{\frac{1}{\rho_{m}s_{11}^{H}(1-\sigma_{m}^{2})}}$ is the sound velocity of Terfenol-D along the radial direction. Under radial harmonic vibration, the displacement $u_{m}$ can be represented as the phasor ${\dot{u}}_{m}$ and Equation (5) can be transformed into its phasor expression as

(6) $$\;\frac{\partial^{2}{\dot{u}}_{m}}{\partial r^{2}}+\frac{1}{r}\frac{\partial {\dot{u}}_{m}}{\partial r}-\frac{{\dot{u}}_{m}}{r^{2}}+k_{m}^{2}{\dot{u}}_{m}=0,$$

where $k_{m}=\frac{\omega }{\upsilon_{m}}$ is the wave number and ω is the angular frequency of the AC magnetic field. By dividing $k_{m}^{2}$, Equation (6) is written as the standard form of the first order Bessel’s differential equation

(7) $$\;\frac{\partial^{2}{\dot{u}}_{m}}{\partial {\left(k_{m}r\right)}^{2}}+\frac{1}{k_{m}r}\frac{\partial {\dot{u}}_{m}}{\partial (k_{m}r)}+[1-\frac{1}{{\left(k_{m}r\right)}^{2}}]{\dot{u}}_{m}=0,$$

whose general solution is

(8) $$\;{\dot{u}}_{m}=AJ_{1}(k_{m}r)+BY_{1}(k_{m}r),$$

where $J_{1}(k_{m}r)$ is the first order Bessel function of the first kind, $Y_{1}(k_{m}r)$ is the first order Bessel function of the second kind, and *A* and *B* are the parameters determined by the boundary conditions. It is obvious that there is no displacement at the center *r* = 0, where the corresponding $Y_{1}(k_{m}r)$ tends to infinite, so the general solution is not available unless *B* = 0. Then,

(9) $$\;{\dot{u}}_{m}=AJ_{1}(k_{m}r).$$

According to the equilibrium of forces, by applying Equations (2a) and (4), the external force exerted on the circumferential surface of Terfenol-D satisfies

(10) $$\;{\dot{F}}_{m}+\frac{A_{m}}{s_{11}^{H}(1-\sigma_{m}^{2})}{\left(\frac{\partial {\dot{u}}_{m}}{\partial r}+\sigma_{m}\frac{{\dot{u}}_{m}}{r}\right)|}_{r=a}-N_{m}{\dot{H}}_{3}=0,$$

where $A_{m}=2\pi at_{m}$ is the circumferential surface area of Terfenol-D, $N_{m}=\frac{2\pi at_{m}d_{31,m}}{s_{11}^{H}(1-\sigma_{m})}$ is the magnetoelastic coupling factor, *a* and *t*_m_ are the radius and thickness of Terfenol-D, respectively. By applying the following recursion formula of the Bessel function of the first kind

(11) $$\;{J^{\prime }}_{1}(x)=J_{0}(x)-\frac{J_{1}(x)}{x},$$

Equation (9) can be transformed into

(12) $$\;\frac{\partial {\dot{u}}_{m}}{\partial r}=A_{m}[k_{m}J_{0}(k_{m}r)-\frac{1}{r}J_{1}(k_{m}r)].$$

**Figure 2:**
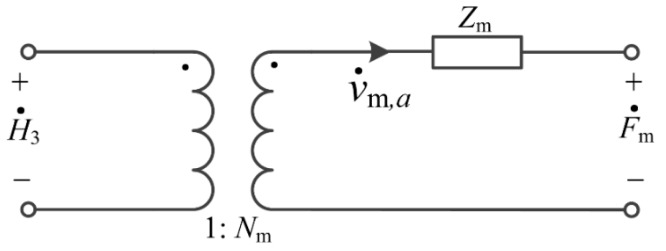
The equivalent circuit of the Terfenol-D layer.

Then, the radial vibration velocity of Terfenol-D layer at the circumferential edge could be represented as $v_{m,a}={\frac{\partial u_{m}}{\partial t}|}_{r=a}$. By substituting Equation (12) into Equation (10) and applying ${\dot{v}}_{m,a}=j\omega {\dot{u}}_{m,a}$, the magnetoelastic coupling equation of Terfenol-D is given as

(13) $${\dot{F}}_{m}=-j\rho_{m}\upsilon_{m}A_{m}[-\frac{J_{0}(k_{m}a)}{J1(k_{m}a)}+\frac{1-\sigma_{m}}{k_{m}a}]{\dot{v}}_{m,a}+N_{m}{\dot{H}}_{3},$$

where ${\dot{F}}_{m}$ and ${\dot{H}}_{3}$ can be analogous to mechanical voltages in an equivalent circuit, ${\dot{v}}_{m,a}$ can be analogous to mechanical current and $Z_{m}=j\rho_{m}\upsilon_{m}A_{m}[-\frac{J_{0}(k_{m}a)}{J1(k_{m}a)}+\frac{1-\sigma_{m}}{k_{m}a}]$ can be analogous to mechanical impedance. Thus, the equivalent circuit of the Terfenol-D layer is shown in Figure 2.

The expression for the Bessel function of the first kind is

(14a) $$\;J_{0}(x)=1-{\left(\frac{x}{2}\right)}^{2}+\frac{1}{{\left(2!\right)}^{2}}{\left(\frac{x}{2}\right)}^{4}-\frac{1}{{\left(3!\right)}^{2}}{\left(\frac{x}{2}\right)}^{6}+\cdots ,$$

(14b) $$J_{1}(x)=\frac{x}{2}-\frac{1}{2!}{\left(\frac{x}{2}\right)}^{3}+\frac{1}{2!3!}{\left(\frac{x}{2}\right)}^{5}-\cdots .$$

When *x* is relative small, i.e., *x* tends to zero, by reserving the first several terms, the Bessel function can be approximate to

(15) $$J_{n}(x)\approx {\left(\frac{x}{2}\right)}^{n}\frac{1}{\Gamma (n+1)}={\left(\frac{x}{2}\right)}^{n}\frac{1}{n!}.$$

Under the low frequency AC magnetic field drive, taking *f* = 50 Hz as an example, $k_{m}a=\frac{\omega }{\upsilon_{m}}a=2\pi fa\sqrt{\rho_{m}s_{11}^{H}(1-{\sigma_{m}}^{2})}=3.24\times 10^{-3}$ is very small for Terfenol-D disk, so $\frac{J_{0}(k_{m}a)}{J_{1}(k_{m}a)}=\frac{1}{\frac{k_{m}a}{2}}=\frac{2}{k_{m}a}$ and the mechanical impedance of the Terfenol-D layer can be simplified to

(16) $$Z_{m}=j\rho_{m}\upsilon_{m}A_{m}(\frac{1+\sigma_{m}}{k_{m}a}).$$

### 2.2. Equivalent Circuit of Piezoelectric Layer

The top and bottom surfaces of PZT are covered with conductive silver electrodes. When the PZT layer undergoes the forced radial symmetrical vibration, the constitutive equations consist of nonzero terms for PZT with ∞ mm symmetry are

(17a) $$S_{rp}=s_{11}^{E}T_{rp}+s_{12}^{E}T_{\theta p}+d_{31,p}E_{z},$$

(17b) $$S_{\theta p}=s_{12}^{E}T_{rp}+s_{11}^{E}T_{\theta p}+d_{31,p}E_{z},$$

(17c) $$D_{z}=d_{31,p}T_{rp}+d_{31,p}T_{\theta p}+\varepsilon_{33}^{T}E_{z}.$$

where the subscript p denotes the piezoelectric layer, $S_{rp}$ and $S_{\theta p}$ are the strain along the radial and azimuth direction, respectively. $T_{rp}$ and $T_{\theta p}$ are the stress along the radial and azimuth direction, respectively. $E_{z}$ and $D_{z}$ are the electric field and dielectric displacement along the thickness direction. $s_{11}^{E}$ and $s_{12}^{E}$ are the compliance coefficients at constant *E*. $d_{31,p}$ is the transverse piezoelectric coefficient. $\varepsilon_{33}^{T}$ is the permeability at constant stress along the axial direction. The constitutive equations can be rewritten in terms of the stress and dielectric displacement as

(18a) $$T_{rp}=\frac{1}{s_{11}^{E}(1-\sigma^{2})}(S_{rp}+\sigma_{p}S_{\theta p})-\frac{d_{31,p}}{s_{11}^{E}(1-\sigma^{2})}E_{z},$$

(18b) $$T_{\theta p}=\frac{1}{s_{11}^{E}(1-\sigma^{2})}(S_{\theta p}+\sigma_{p}S_{rp})-\frac{d_{31,p}}{s_{11}^{E}(1-\sigma^{2})}E_{z},$$

(18c) $$D_{z}=d_{31,p}(T_{rp}+T_{\theta p})+\varepsilon_{33}^{T}E_{z},$$

where $\sigma_{p}=-\frac{s_{12}^{E}}{s_{11}^{E}}$ is the Possion ratio of PZT. According to the Newton’s second law, the radial equation of motion for the PZT layer is

(19) $$\frac{\partial T_{rp}}{\partial r}+\frac{T_{rp}-T_{\theta p}}{r}=\rho_{p}\frac{\partial^{2}u_{rp}}{\partial t^{2}},$$

where $\rho_{p}$ and $u_{rp}$ is the density and displacement of PZT layer. By substituting Equations (18a) and (18b) into Equation (19), considering the surface of the electrode is equipotential, i.e., $\partial E_{z}/\partial r=0$, and using the definition of strain

(20) $$S_{rp}=\frac{\partial u_{rp}}{\partial r},S_{\theta p}=\frac{u_{rp}}{r},$$

the radial equation of motion can be rewritten as (*u*_rp_ is simplified as *u*_p_ for convenience)

(21) $$\frac{\partial^{2}u_{p}}{\partial r^{2}}+\frac{1}{r}\frac{\partial u_{p}}{\partial r}-\frac{u_{p}}{r^{2}}=\frac{1}{\upsilon_{p}^{2}}\frac{\partial^{2}u_{p}}{\partial t^{2}},$$

where $\upsilon_{p}=\sqrt{\frac{1}{\rho_{p}s_{11}^{E}(1-\sigma_{p}^{2})}}$ is the sound velocity of PZT along the radial direction. Under radial harmonic vibration, the displacement $u_{p}$ can be represented as the phasor ${\dot{u}}_{p}$ and Equation (21) can be transformed into its phasor expression as

(22) $$\frac{\partial^{2}{\dot{u}}_{p}}{\partial r^{2}}+\frac{1}{r}\frac{\partial {\dot{u}}_{p}}{\partial r}-\frac{{\dot{u}}_{p}}{r^{2}}+k_{p}^{2}{\dot{u}}_{p}=0,$$

where $k_{p}=\frac{\omega }{\upsilon_{p}}$ is the wave number and ω is the angular frequency of AC magnetic field. By dividing $k_{p}^{2}$, Equation (22) is written as the standard form of the first order Bessel’s differential equation

(23) $$\frac{\partial^{2}{\dot{u}}_{p}}{\partial {\left(k_{p}r\right)}^{2}}+\frac{1}{k_{p}r}\frac{\partial {\dot{u}}_{p}}{\partial (k_{p}r)}+[1-\frac{1}{{\left(k_{p}r\right)}^{2}}]{\dot{u}}_{p}=0,$$

whose general solution is

(24) $${\dot{u}}_{p}=CJ_{1}(k_{p}r)+DY_{1}(k_{p}r),$$

where $J_{1}(k_{p}r)$ is the first order Bessel function of the first kind, $Y_{1}(k_{p}r)$ is the first order Bessel function of the second kind, *C* and *D* is the parameters determined by the boundary conditions. It is obvious that there is no displacement at the center r=0, where the corresponding $Y_{1}(k_{m}r)$ tends to infinite, so the general solution is not available unless D=0. Then

(25) $${\dot{u}}_{p}=CJ_{1}(k_{p}r).$$

According to the equilibrium of forces, by applying Equations (18a) and (20), the external force exerted on the circumferential surface of Terfenol-D satisfies

(26) $${\dot{F}}_{p}+\frac{A_{p}Y_{0}^{E}}{\left(1-\sigma_{p}^{2}\right)}{\left(\frac{\partial {\dot{u}}_{p}}{\partial r}+\sigma_{p}\frac{{\dot{u}}_{p}}{r}\right)|}_{r=a}-N_{p}{\dot{V}}_{3}=0,$$

where $A_{p}=2\pi at_{p}$ is the circumferential surface area of PZT layer, $N_{p}=\frac{2\pi ad_{31,p}}{s_{11}^{E}(1-\sigma_{p})}$ is the electromechanical coupling factor, and ${\dot{V}}_{3}={\dot{E}}_{z}t_{p}$ is the induced voltage across the PZT. *a* and $t_{p}$ are the radius and thickness of PZT, respectively. By applying the following recursion formula of the Bessel function of the first kind

(27) $${J^{\prime }}_{1}(x)=J_{0}(x)-\frac{J_{1}(x)}{x},$$

Equation (25) can be transformed into

(28) $$\frac{\partial u_{p}}{\partial r}=A_{p}[k_{p}J_{0}(k_{p}r)-\frac{1}{r}J_{1}(k_{p}r)].$$

Then, the radial vibration velocity of PZT layer at the circumferential edge could be represented as $v_{p,a}={\frac{\partial u_{p}}{\partial t}|}_{r=a}$. By substituting Equation (28) into Equation (26) and applying ${\dot{v}}_{p,a}=j\omega {\dot{u}}_{p,a}$, the electromechanical coupling equation of PZT is given as

(29) $$-{\dot{F}}_{p}=j\rho_{p}\upsilon_{p}A_{p}[-\frac{J_{0}(k_{p}a)}{J_{1}(k_{p}a)}+\frac{1-\sigma_{p}}{k_{p}a}]{\dot{v}}_{p,a}-N_{p}{\dot{V}}_{3}.$$

Furthermore, by substituting Equations (18a) and (18b) into Equation (18c) and applying Equation (20), the dielectric displacement can be solved as

(30) $$\begin{array}{cc} D_{z}= & \left[\varepsilon_{33}^{T}-\frac{2d_{31}^{2}}{s_{11}^{E}(1-\sigma_{p})}]E_{z}+\frac{d_{31,p}}{s_{11}^{E}(1-\sigma_{p})}(S_{rp}+S_{\theta p}\right)\\ = & \;\varepsilon_{33}^{T}(1-k_{p}^{2})E_{z}+\frac{d_{31,p}}{s_{11}^{E}(1-\sigma_{p})}(\frac{\partial u_{rp}}{\partial r}+\frac{u_{rp}}{r}) \end{array}.$$

Integrating Equation (30) over the area of the electrode surface, the electric charges induced on the electrode surface can be determined as

(31) $$\begin{array}{lll} Q & = & \int_{0}^{2\pi }\int_{0}^{a}D_{z}rdrd\theta \\ & = & \;\int_{0}^{2\pi }\int_{0}^{a}\left[\varepsilon_{33}^{T}(1-k_{p}^{2})E_{z}+\frac{d_{31,p}}{s_{11}^{E}(1-\sigma_{p})}(\frac{\partial u_{rp}}{\partial r}+\frac{u_{rp}}{r}\right)]rdrd\theta \\ & = & \;\pi a^{2}\varepsilon_{33}^{T}(1-k_{p}^{2})\frac{V_{3}}{t_{p}}+\frac{2\pi ad_{31,p}}{s_{11}^{E}(1-\sigma_{p})}u_{p,a} \end{array}.$$

**Figure 3:**
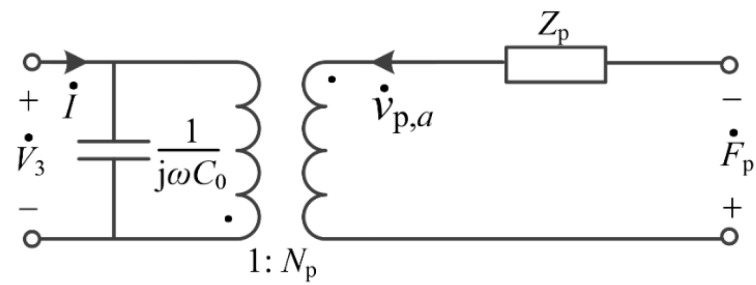
The equivalent circuit of the PZT (Pb(Zr,Ti)O_3_) layer.

Under harmonic vibration, the electric charges *Q* induced by the piezoelectric layer and the current *I* flowing into the piezoelectric layer can be expressed as phasors $\dot{Q}$ and $\dot{I}$, respectively. The relationship between them is

(32) $$\begin{array}{ll} \dot{I} & =j\omega \dot{Q}\\ & =j\omega \;[\pi a^{2}\varepsilon_{33}^{T}(1-k_{p}^{2})\frac{{\dot{V}}_{3}}{t_{p}}+\frac{2\pi ad_{31,p}}{s_{11}^{E}(1-\sigma_{p})}{\dot{u}}_{p,a}]\\ & =\;j\omega \;\frac{\pi a^{2}\varepsilon_{33}^{T}(1-k_{p}^{2})}{t_{p}}{\dot{V}}_{3}+j\omega \;{\dot{u}}_{p,a}\frac{2\pi ad_{31,p}}{s_{11}^{E}(1-\sigma_{p})}\\ & =\;j\omega \;C_{0}{\dot{V}}_{3}+N_{p}{\dot{v}}_{p,a} \end{array}.$$

where $C_{0}=\frac{\pi a^{2}\varepsilon_{33}^{T}(1-k_{p}^{2})}{t_{p}}$ is static capacitance of the PZT layer. In Equations (29) and (32), ${\dot{F}}_{p}$ and ${\dot{v}}_{p,a}$ can be analogous to mechanical voltage and mechanical current in an electric circuit, $Z_{p}=j\rho_{p}\upsilon_{p}A_{p}[-\frac{J_{0}(k_{p}a)}{J_{1}(k_{p}a)}+\frac{1-\sigma_{p}}{k_{p}a}]$ can be analogous to mechanical impedance, and ${\dot{V}}_{3}$ and $\dot{I}$ are realistic electrical voltage and current, respectively. Thus, the equivalent circuit of the PZT layer is shown in Figure 3.

Under the low frequency AC magnetic field drive, taking *f* = 50 Hz as an example, $k_{p}a=\frac{\omega }{\upsilon_{p}}a=2\pi fa\sqrt{\rho s_{11}^{E}(1-\sigma^{2})}=1.04\times 10^{-3}$ is very small for PZT-5 disk, so $\frac{J_{0}(k_{p}a)}{J_{1}(k_{p}a)}=\frac{1}{\frac{k_{p}a}{2}}=\frac{2}{k_{p}a}$ and the mechanical impedance of the PZT layer can be simplified to

(33) $$Z_{p}=-j\rho_{p}\upsilon_{p}A_{p}(\frac{1+\sigma_{p}}{k_{p}a}).$$

### 2.3. Introducing Interface Coupling Factor

The expression for the interface coupling factor in the long-type ME laminate composite proposed by Bichurin et al. is $k_{c}=(S_{1p}-S_{10,p})/(S_{1m}-S_{10,p})$ [17], where $S_{1p}$ and $S_{1m}$ are the longitudinal strains of the piezoelectric and magnetostrictive layer, respectively, and $S_{10,p}$ is the longitudinal strain of piezoelectric layer with no friction between the layers. However, the major deficiencies of this interface coupling factor are as follows: (i) $S_{10,p}$ could hardly be measured or calculated, and the expression for $k_{c}$ lacks a simple and straightforward physical meaning; and (ii) the definition of $k_{c}$ proposed by Bichurin et al. is only applicable for the static or quasi-static condition in the elastic model. If it is applied to couple the equivalent circuits of magnetostrictive and piezoelectric layers for the dynamic condition, there would be two unnecessary mechanical current sources in the combined equivalent circuit of the whole laminate composite due to the existence of $S_{10,p}$, resulting in a much more complicated expression for the ME coefficient. Therefore, a new interface coupling factor which physically reflects the strain transfer between the phases should be proposed in the equivalent circuit model for quasi-static and dynamic conditions. Considering the radial strain is

(34) $$S_{r}=\frac{\partial u_{r}}{\partial r}=\frac{u_{r}(a)-u_{r}(0)}{a}=\frac{\int v_{r}(a)dt-\int v_{r}(0)dt}{a},$$

whose phasor form is

(35) $${\dot{S}}_{r}=\frac{{\dot{v}}_{r}(a)-{\dot{v}}_{r}(0)}{j\omega a},$$

without loss of generality, let

(36) $$k_{c}=\frac{{\dot{S}}_{rp}}{{\dot{S}}_{rm}}=\frac{{\dot{v}}_{rp}(a)-{\dot{v}}_{rp}(0)}{{\dot{v}}_{rm}(a)-{\dot{v}}_{rm}(0)}=\frac{{\dot{v}}_{p,a}}{{\dot{v}}_{m,a}}.$$

When the interface coupling factor $k_{c}=1$, i.e., $S_{rp}=S_{rm}$, the strains of two phase material are equal, which means the strain of magnetostrictive layer is totally transferred to the piezoelectric layer through the epoxy resin adhesive and the interface coupling between them is ideal. When $k_{c}=0$, i.e., $S_{rp}=0$, the strain of piezoelectric layer is always zero no matter how the magnetostrictive layers shrink and stretch, which means the strain of magnetostrictive layer could hardly be transferred to the piezoelectric layer through the epoxy resin adhesive and there is no interface coupling or friction between them. When *k_c_* is between zero and one, the strain of magnetostrictive layer is partially transferred to the piezoelectric layer and the actual coupling condition is quantitatively represented by *k_c_*.

Under free boundary condition, the radial vibration velocity at the circumferential edge ${\dot{v}}_{m,a}$ and ${\dot{v}}_{p,a}$ are the mechanical current on the secondary side of the ideal transformer in Figure 2 and Figure 3. By applying Equation (36), the equivalent circuits of the two phases could be coupled through an ideal transformer with a turn ratio *k_c_*, which transfers the radial strain (or vibration) from the magnetostrictive layer to the piezoelectric layer in the direct ME effect.

Meanwhile, under the mechanical boundary condition that the resultant external force exerted on the ME laminate composite is zero (i.e., ${\dot{F}}_{m}+{\dot{F}}_{p}=0$) in the unclamped harmonic vibration, and under the electrical boundary condition that the piezoelectric layer could be regarded as open-circuited (i.e., the dielectric displacement $D_{z}=0$) in measuring its output, the ME laminate composite satisfies

(37a) $$-Z_{m}{\dot{v}}_{m,a}+N_{m}{\dot{H}}_{3}-Z_{p}{\dot{v}}_{p,a}+N_{p}{\dot{V}}_{3}=0,$$

(37b) $$\dot{I}=\;j\omega C_{0}{\dot{V}}_{3}+N_{p}{\dot{v}}_{p,a}=0,$$

(37c) $${\dot{v}}_{p,a}=k_{c}{\dot{v}}_{m,a}.$$

**Figure 4:**
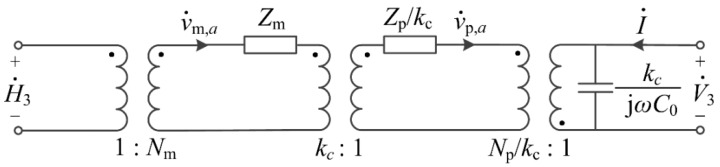
The equivalent circuit of the T-T mode disk-type Terfenol-D/PZT laminate composite.

From the equations above, the equivalent circuit of the T-T mode disk-type Terfenol-D/PZT laminate composite could be developed, as shown in Figure 4. The applied small AC magnetic field drive ${\dot{H}}_{3}$ on the Terfenol-D layer is eventually transformed into the induced electrical voltage ${\dot{V}}_{3}$ on the PZT layer through three ideal transformers whose turn ratios are the magnetoelastic coupling factor, the newly proposed interface coupling factor and the electromechanical coupling factor, respectively. Comparing with the equivalent circuit derived by Dong [22], this is a more physical and precise model by introducing the interface coupling factor, which is not discussed in Dong’s model. It should also be mentioned that this is a version observed from the Terfenol-D layer, in which the mechanical current in the main circuit loop is ${\dot{v}}_{m,a}$, the mechanical impedance, the electromechanical coupling factor and static capacitance of PZT layer is converted to the primary side of the ideal transformer whose turn ratio is *k_c_*.

According to the magneto-elasto-electric equivalent circuit, the ME voltage coefficient of T-T mode disk-type ME laminate composite could be obtained. By applying the voltage divider rule, the ratio of the output mechanical voltage $N_{p}{\dot{V}}_{3}$ to the exciting mechanical voltage $N_{m}{\dot{H}}_{3}$ is

(38) $$\left|\frac{N_{p}V_{3}}{N_{m}H_{3}}\right|=\left|\frac{\frac{N_{p}^{2}k_{c}}{j\omega C_{0}}}{Z_{m}+k_{c}Z_{p}+\frac{N_{p}^{2}k_{c}}{j\omega C_{0}}}\right|.$$

By substituting the parameters into Equation (38), the ME voltage coefficient of T-T mode disk-type ME laminate composite could be derived as

(39) $$\alpha_{V}={\left|\frac{dV_{3}}{dH_{3}}\right|}_{T-T}=\frac{2n(1-n)t_{\mathrm{total}}d_{31,m}g_{31,p}k_{c}}{ns_{11}^{E}(1-\sigma_{p})(1-k_{p}^{2})+(1-n)k_{c}s_{11}^{H}(1-\sigma_{m})},$$

where $n=\frac{t_{m}}{t_{\mathrm{total}}}=\frac{t_{m}}{t_{m}+t_{p}}$ is the thickness ratio, which means the ratio of the thickness of Terfenol-D layers $t_{m}$ to the total thickness of the ME laminate $t_{\mathrm{total}}$. The numerator of Equation (39) is a quadratic function of *n*, so it could be concluded that there is an optimum thickness ratio $n_{\mathrm{optim}}$ corresponds to the maximum ME voltage coefficient. By letting $\delta \alpha_{V}/\delta n=0$, the value of the optimum thickness ratio could be solved as

(40) $$\begin{array}{l} n_{\mathrm{optim}}=\frac{1}{1+\sqrt{\frac{s_{11}^{E}(1-\sigma_{p})}{k_{c}s_{11}^{H}(1-\sigma_{m})}(1-k_{p}^{2})}}\\ \;=\frac{1}{1+\sqrt{\frac{s_{11}^{E}(1-\sigma_{p})}{k_{c}s_{11}^{H}(1-\sigma_{m})}(1-\frac{2}{1-\sigma_{p}}\frac{d_{31,p}^{2}}{\varepsilon_{33}^{T}s_{11}^{E}})}}\\ \;=\frac{1}{1+\sqrt{\frac{s_{11}^{E}(1-\sigma_{p})}{k_{c}s_{11}^{H}(1-\sigma_{m})}\frac{\varepsilon_{33}^{S}}{\varepsilon_{33}^{T}}}} \end{array}.$$

## 3. Sample Fabrication and Experimental Setup

The Terfenol-D disks (Central Iron and Steel Research Institute, Beijing, China) oriented along the thickness direction were machined to the dimension of φ20 × 2 mm. In order to explore the relationship between the ME voltage coefficient and thickness ratio and verify the existence of the optimum thickness ratio, the transversely polarized PZT-5 disks (Sunnytec Company, Suzhou, China) covered by silver electrodes on the top and bottom surfaces were machined to eleven sets of dimensions and the diameters were always kept as 20 mm. The parameters of Terfenol-D and PZT-5 are listed in Table 1.

Two Terfenol-D disks and one PZT-5 disk were laminated as a sandwich structure by epoxy resin adhesive (Eccobond 45 BLK, Emerson & Cuming, Germantown, WI, USA) and cured at 85 °C for 6 h under mechanical load for a better bonding. The interface coupling factor, i.e., the strain transfer efficiency between the layers, could be influenced by the adhesive bonding condition, which is related to the temperature and pressure. Therefore in the sample fabrication (not in the test), two sets of weights, 100 g and 500 g were placed on the top of ME laminates with the same thickness ratio to provide different mechanical pressure for comparison. All the fabricated samples were tagged with numbers for convenience and their configurations are listed in Table 2 and Table 3.

**Figure 5:**
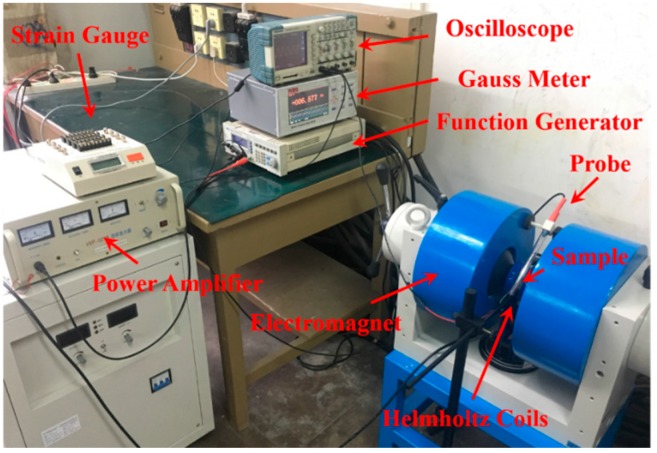
The experimental setup for ME measurement system.

Figure 5 shows the experimental setup for measuring the ME voltage coefficient. An electromagnet (EM5, East Changing Technologies, Inc., Beijing, China) is used to supply a DC bias magnetic field *H*_bias_ up to 5000 Oe for the ME laminate. A digital function generator (SP 1212B) and a power amplifier (HVP-1070B, Foneng Tech, Inc., Nanjing, China) supply the drive signal to a pair of Helmholtz coils to generate a small AC magnetic field *H*_ac_ in parallel superimposed on *H*_bias_, ranging from 0 to 10 Oe at *f* = 50 Hz, which is probed by a Gauss meter (Model 905, Honor Top Magnetic Tech Co., Ltd., Qingdao, China). The induced voltage of PZT layer is observed by an oscilloscope (Tektronix TPS 2014). Because the Terfenol-D layers can be regarded as conductive, the output voltage between the top and bottom surfaces of the laminate composite equals the induced voltage between the surfaces of the PZT layer because of its transverse polarization. The outlet conducting wires are soldered on the top and bottom surfaces of whole ME laminate for convenience.

## 4. Results and Discussions

### 4.1. Magnetostriction and Piezomagnetic Coefficient

From the theoretical expression Equation (39), it can be seen that the ME voltage coefficient *α*_v_ for T-T mode is proportional to the piezomagnetic coefficient *d*_31,m_, which is a function of the DC bias magnetic field. Considering *d*_31,m_ is the derivative of *λ*_31,m_ with respect to the DC bias magnetic field, the magnetostriction of Terfenol-D should be discussed firstly. The magnetostriction of Terfenol-D is measured by a resistance strain gauge and a Wheatstone bridge (YJZ-8, TRX Co., Ltd., Beijing, China). For the convenience of measurement, a longitudinally magnetized long-type Terfenol-D machined from the commercial Terfenol-D rod is applied, instead of a transversely magnetized disk-type Terfenol-D.

**Figure 6:**
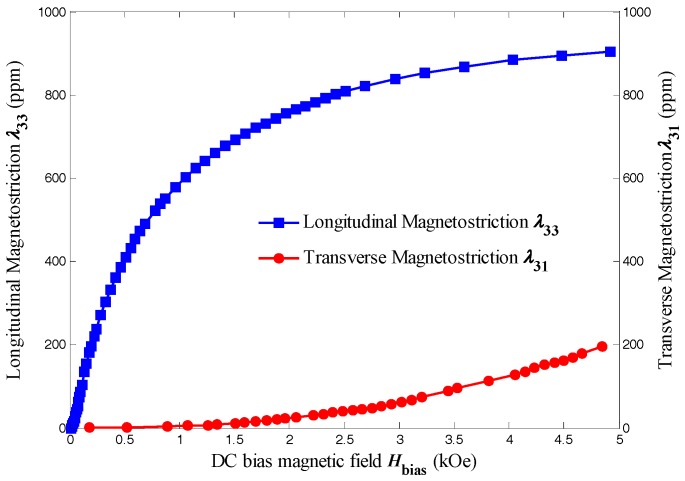
The longitudinal magnetostriction *λ*_33_ and transverse magnetostriction *λ*_31_ of Terfenol-D as a function of the DC bias magnetic field *H*_bias_ at room temperature.

**Figure 7:**
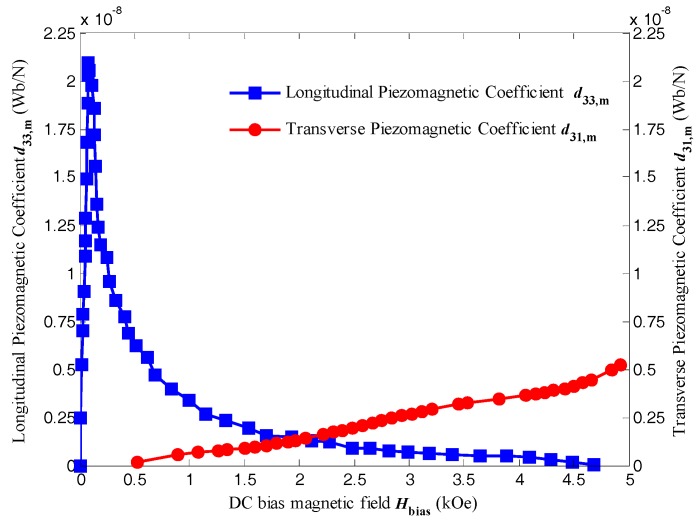
The longitudinal piezomagnetic coefficient *d*_33,m_ and transverse piezomagnetic coefficient *d*_31,m_ of Terfenol-D as a function of the DC bias magnetic field *H*_bias_, which are the respective slopes calculated from the corresponding magnetostriction curves in Figure 6.

Figure 6 shows the longitudinal magnetostriction *λ*_33_ and transverse magnetostriction *λ*_31_ of Terfenol-D as a function of the DC bias magnetic field *H*_bias_ at room temperature measured by a strain gauge method. By calculating the slope of the measured *λ*-*H*_bias_ curves using a six-order polynomial curve fitting, the longitudinal and transverse piezomagnetic coefficient of Terfenol-D as a function of the DC bias magnetic field *H*_bias_ is illustrated in Figure 7.

It should be noticed that the transverse magnetostriction is negative when *H*_bias_ is applied along the thickness direction of the Terfenol-D bar, but it is illustrated as positive for comparison. It can be obviously seen that the longitudinal magnetostriction is more than four times greater than that of the transverse magnetostriction with DC bias magnetic field ranging from 0 to 5000 Oe. Upon increasing *H*_bias_, the longitudinal magnetostriction almost reaches its saturation near 900 ppm at 3000 Oe, whereas the transverse magnetostriction increases steadily without showing the sign of saturation. The differences in the magnitude and curve tendency could be attributed to the demagnetization field. When *H*_bias_ is applied along the longitudinal direction in which the demagnetization factor of the Terfernol-D bar is small, the net magnetic flux is large and the induced strain is saturated at lower magnetic field while along the thickness direction in which the demagnetization factor is relative large, the net magnetic flux is small and the induced strain could hardly reach saturation. Although the longitudinal magnetostriction is much higher than the transverse one, their corresponding piezomagnetic coefficient is more comparable. *d*_33,m_ reaches its maximum value higher than 2 × 10^−8^ Wb/N under 100 Oe, but drops rapidly to lower than 2.5 × 10^−9^ Wb/N under 1000 Oe or more. However, *d*_31,m_ keeps rising and even exceeds *d*_33,m_ under the magnetic field higher than 2000 Oe, which reveals an advantage of T-T mode ME laminate. Considering the ME voltage coefficient is proportional to the piezomagnetic coefficient, T-T mode disk-type ME laminate shows a potential for providing higher ME coefficient in the applications as sensors and actuators under large DC magnetic field environment in comparison to other configurations, such as L-T mode or L-L mode long-type ME laminate.

### 4.2. ME Voltage Coefficient

**Figure 8:**
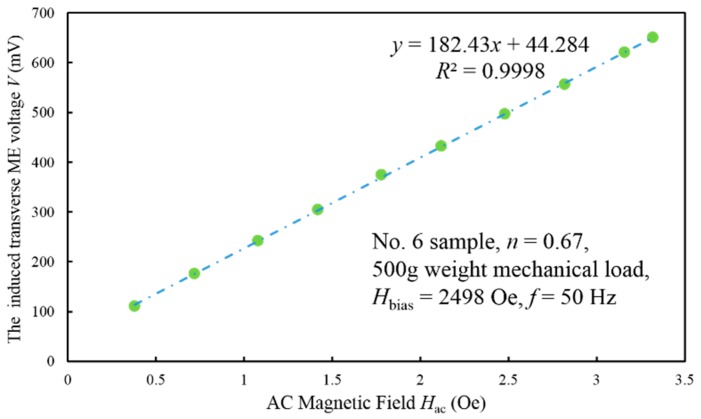
The induced transverse ME voltage as a function of the AC magnetic field *H*_ac_ for sample No. 6 at *H*_bias_ = 2498 Oe and *f* = 50 Hz. The green scattered points represent the measured data under 500 g pre-mechanical load and the blue dash-dot line is the trend line derived from the linear regression analysis. The linear regression equation and the corresponding correlation coefficient *R* beside the line illustrate the degree of linearity.

Figure 8 shows the induced transverse ME voltage as a function of the AC magnetic field *H*_ac_ for sample No. 6 at *H*_bias_ = 2498 Oe and *f* =50 Hz. The scattered points represent the measured data and the dash-dot line is the trend line derived from the linear regression analysis based on them. The linear regression equation and the corresponding correlation coefficient *R* beside the line illustrate the degree of linearity. It can be clearly seen that the transverse ME voltage varies linearly with the AC magnetic field over the range 0.5–3.5 Oe. Therefore, the slope of the trend line is just the transverse ME voltage coefficient, i.e., *α*_v_ = 0.182 V/Oe in this condition. It also should be noticed that the linear regression equation contains an intercept term, which indicates the ME laminate induces voltage even under *H*_ac_ = 0. This interference could be attributed to the constant ambient stray magnetic field.

**Figure 9:**
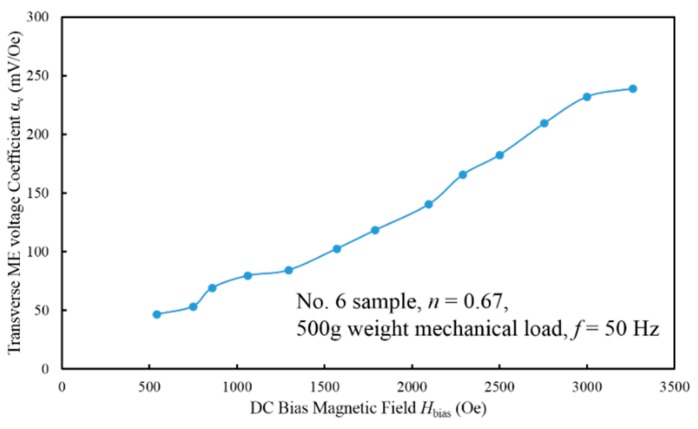
The induced transverse ME voltage coefficient as a function of the DC bias magnetic field for sample No. 6 at *f* = 50 Hz.

By varying the DC bias magnetic field, the variation of the transverse ME voltage coefficient as a function of the DC bias magnetic field for sample No. 6 could be measured, as shown in Figure 9. The transverse ME voltage coefficient increases with the DC bias magnetic field steadily and reaches to 250 mV/Oe in the vicinity of *H*_bias_ = 3000 Oe. Through the comparison with Figure 7, it indicates that the *α*_v_-*H*_bias_ curve approximately follows the tendency of *d*_31,m_-*H*_bias_ curve over the range from 0 to 3000 Oe. This experimentally reflects the fact that the transverse ME voltage *α*_v_ is proportional to *d*_31,m_ while the other parameters in Equation (39), especially the thickness ratio *n* and the interface coupling factor *k*_c_, keeps constant. Another significant conclusion from Figure 9 is that the expression for *α*_v_-*H*_bias_ curve is a monotone function in a wide range of *H*_bias_ from 0 to 3000 Oe, providing the potential for T-T mode disk-type ME laminate composite to measure a large DC current or magnetic field under a constant AC magnetic field drive [29]. The related details are discussed in our future research paper.

### 4.3. Interface Coupling Factor

**Figure 10:**
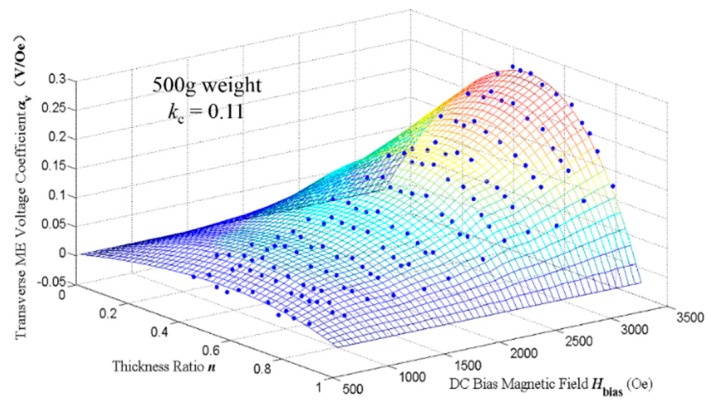
The measured data and surface fitting result in *H*_bias_-*n*-*α*_v_ three-dimensional space for samples No. 1–No. 11. The blue scattered points represent the measured data under 500 g pre-mechanical load and the surface represents the transverse ME voltage coefficient corresponds to *k*_c_ = 0.11.

The interface coupling factor *k*_c_ could be determined by substituting the measured ME voltage coefficient *α*_v_, the piezomagnetic coefficient *d*_31,m_, the thickness ratio *n* and other material parameters into Equation (39). The calculated *k*_c_ of sample No. 6 is listed in Table 4. By ignoring the data corresponds to the too large or small *H*_bias_, it can be seen that the calculated interface coupling factor *k*_c_ is approximate to 0.10–0.13. However, the result for one sample may not reflect the common condition for all the samples in the experiment. There are two steps to find the interface coupling factor that is suitable for all the samples: (i) plot all the measured data in the three-dimensional space formed by *H*_bias_, *n* and *α*_v_; and (ii) based on the least mean square error analysis, fit the measured data with a surface formed by the theoretical ME voltage coefficient in which the interface coupling factor varies in the range from 0.10 to 0.13. The surface with the least mean square error corresponds to the most suitable *k*_c_. Figure 10 shows the measured data and surface fitting result in *H*_bias_-*n*-*α*_v_ three dimensional space for samples No. 1–No. 11, where the blue scattered points represent the measured data and the surface corresponds to *k*_c_ = 0.11. It could be clearly seen that the surface fits the scattered points quite well, indicating the correctness of *k*_c_ = 0.11. In other words, it verifies the reasonability for the introduction of the new defined interface coupling factor in Equation (36). Similarly, the measured data of samples No. 12–No. 22 are dealt with using the same process and the corresponding *k*_c_ = 0.08.

There is a qualitative physical explanation for the fact that the interface coupling factor varies with pre-mechanical load in the sample fabrication. Under 500 g weight pressure, the bonding condition between the layers is relatively rigid, resulting in the relatively much strains transferred from magnetostrictive layer to piezoelectric layer, so the interface coupling factor is larger. Under 100 g weight pressure, the bonding between the layers is weak, the strain transfer is reduced, so the interface coupling factor is relatively small.

However, an interface coupling factor as high as 0.6 has been achieved by Silva et al. [20]. Three epoxy resins, Devcon, M-Bond and Stycast, and three thickness of 28, 52 and 110 μm for the piezoelectric layer were studied. It is their conclusion that an epoxy resin with smaller Young’s modulus and a piezoelectric layer with larger thickness result in a larger ME coefficient, i.e., a larger *k*_c_. It should also be noticed that an optimum thickness for the piezoelectric layer exists at which the ME coefficient reaches its maximum value, since a much larger thickness will lead to the inhomogeneous deformations of the piezoelectric layer, with more deformation at the boundary region with the epoxy resin and lower deformation at the center region of the layer. In our experiment, the Young’s modulus of Eccobond 45 is 0.5 GPa [30], larger than 0.3 GPa of M-Bond 600 and the thickness of piezoelectric layer is over an order of magnitude larger than 110 μm. Therefore, it should be qualitatively reasonable that the interface coupling factor obtained in our experiment is lower than the best 0.6 for the M-Bond bonded ME laminate in [20]. Furthermore, our interface coupling factor is comparable with 0.07 for the Stycast bonded ME laminate.

### 4.4. The Optimum Thickness Ratio

**Figure 11:**
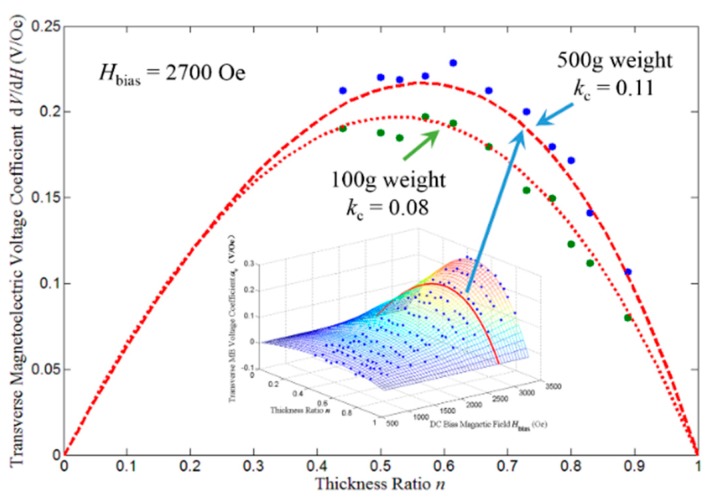
The theoretical and measured transverse ME voltage coefficient as a function of the thickness ratio under 2700 Oe DC bias magnetic field. The scattered blue points represent the measured data under 500 g pre-mechanical load, the scattered green points represent the measured data under 100 g pre-mechanical load, the red dash line represents the theoretical curve for *k*_c_ = 0.11 and the red dotted line represents the theoretical curve for *k*_c_ = 0.08.

According to Equation (40), when k_c_ = 0.11, the optimum thickness ratio for T-T mode disk-type ME laminate is 0.57, while k_c_ = 0.08, the optimum thickness ratio is 0.52. Considering the DC bias magnetic field has no influences on the optimum thickness ratio, the experimental data measured under H_bias_ = 2700 Oe are applied to verify the prediction on the optimum thickness ratio, as shown in Figure 11, where the scattered blue points represent the measured data for k_c_ = 0.11, the scattered green points represent the measured data for k_c_ = 0.08, the red dash line represents the theoretical prediction from Equation (39) for k_c_ = 0.11 and the red dotted line represents the theoretical prediction from Equation (39) for k_c_ = 0.08. It can be seen that the scattered measured points essentially fits the theoretical prediction curve. The measured optimum thickness ratio is 0.61 for k_c_ = 0.11 and 0.56 for k_c_ = 0.08. The measured optimum thickness ratios are both slightly larger than the corresponding theoretical one, but the relationship between them remains unchanged, i.e., the measured optimum thickness ratio for k_c_ = 0.11 is still larger than the one for k_c_ = 0.08.

## 5. Conclusions

The proposed interface coupling factor between the magnetostrictive layer and the piezoelectric layer can effectively represent the coupling extend in a simpler but clearer way.

The respective equivalent circuit models of magnetostrictive and piezoelectric layers can be combined with an ideal transformer whose turn-ratio is just the interface coupling factor. In this way, the equivalent circuit can represent the physical coupling effect between the two phases. 

The more precise expression for the transverse ME voltage coefficient *α*_v_ and the optimum thickness ratio *n*_optim_ can be easily derived from the equivalent circuit mentioned above. A total of 22 sample experiments with different configurations verify the correctness of the equivalent circuit and the derived expression.

The model proposed in this paper is only suitable for the ME laminate composites, in which the magnetostrictive and piezoelectric layers are coupled together by epoxy resin adhesive or other glues. It may not essentially explain the magnetoelectric effects from a physical aspect in the particulate composites, the thin films and other ME composites. However, if we regard the interface coupling factor as a rectify coefficient, it may also explain the discrepancy between the theoretical and experimental results for these composites mentioned above [20,21].

Overall, this modified equivalent circuit model can provide guidance for the design, fabrication and application of the ME laminate based devices, such as current sensor [31], magnetic sensor, energy harvester and wireless energy transfer system [32].

## Figures and Tables

**Table 1:** The parameters of Terfenol-D and PZT-5.

Material	*d*_31,m_ or *g*_31,p_	$s_{11}^{H}$ or $s_{11}^{E}$	σ	*μ*_r_	*k*_p_
Terfenol-D ^1^	2 × 10^−10^ Wb/N	125 × 10^−12^ m^2^/N	0.35	5	
PZT-5 ^2^	10 × 10^−3^ Vm/N	13.5 × 10^−12^ m^2^/N	0.36	1	0.62

^1^ Cited from Central Iron and Steel Research Institute, China; ^2^ Cited from Sunnytec Company, China.

**Table 2:** The configuration of ME laminate sample under 500 g weight mechanical load.

Sample Number	1	2	3	4	5	6	7	8	9	10	11
Terfenol-D (mm)	2	2	2	2	2	2	2	2	2	2	2
PZT-5 (mm)	0.5	0.8	1	1.2	1.5	2	2.5	3	3.5	4	5
Thickness ratio *n*	0.89	0.83	0.8	0.77	0.73	0.67	0.615	0.57	0.53	0.5	0.44
Pre-mechanical load	500 g weight										

**Table 3:** The configuration of ME laminate sample under 100 g weight mechanical load.

Sample Number	12	13	14	15	16	17	18	19	20	21	22
Terfenol-D (mm)	2	2	2	2	2	2	2	2	2	2	2
PZT-5 (mm)	0.5	0.8	1	1.2	1.5	2	2.5	3	3.5	4	5
Thickness ratio *n*	0.89	0.83	0.8	0.77	0.73	0.67	0.615	0.57	0.53	0.5	0.44
Pre-mechanical load	100 g weight										

**Table 4:** The calculated interface coupling factor *k*_c_ of sample No. 6.

Parameters	Values												
*H*_bias_ (Oe)	541	748	857	1059	1294	1569	1787	2095	2288	2498	2754	2998	3259
*d*_31,m_ (10^−10^ A/m)	0.9	1.4	1.7	1.92	2.05	2.60	2.90	3.70	4.54	4.85	5.70	6.60	7.40
α_v_ (mV/Oe)	46.57	53.32	69.03	79.51	84.22	102.52	118.21	140.39	165.58	182.43	209.43	232.11	239.10
Calculated *k*_c_	0.224	0.113	0.129	0.134	0.131	0.122	0.129	0.113	0.106	0.111	0.106	0.099	0.086
